# The metabolic control of DNA replication: mechanism and function

**DOI:** 10.1098/rsob.230220

**Published:** 2023-08-16

**Authors:** Panos Soultanas, Laurent Janniere

**Affiliations:** ^1^ Biodiscovery Institute, School of Chemistry, University of Nottingham, University Park, Nottingham NG7 2RD, UK; ^2^ Génomique Métabolique, Genoscope, Institut François Jacob, CEA, CNRS, Université Evry, Université Paris-Saclay, 91057 Evry, France

**Keywords:** DNA replication, central carbon metabolism, signalling, cell cycle, allosteric regulation, moonlighting functions

## Abstract

Metabolism and DNA replication are the two most fundamental biological functions in life. The catabolic branch of metabolism breaks down nutrients to produce energy and precursors used by the anabolic branch of metabolism to synthesize macromolecules. DNA replication consumes energy and precursors for faithfully copying genomes, propagating the genetic material from generation to generation. We have exquisite understanding of the mechanisms that underpin and regulate these two biological functions. However, the molecular mechanism coordinating replication to metabolism and its biological function remains mostly unknown. Understanding how and why living organisms respond to fluctuating nutritional stimuli through cell-cycle dynamic changes and reproducibly and distinctly temporalize DNA synthesis in a wide-range of growth conditions is important, with wider implications across all domains of life. After summarizing the seminal studies that founded the concept of the metabolic control of replication, we review data linking metabolism to replication from bacteria to humans. Molecular insights underpinning these links are then presented to propose that the metabolic control of replication uses signalling systems gearing metabolome homeostasis to orchestrate replication temporalization. The remarkable replication phenotypes found in mutants of this control highlight its importance in replication regulation and potentially genetic stability and tumorigenesis.

## Graphical abstract

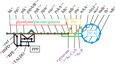

## 1. Background

Complex mechanochemical molecular machines, known as replisomes, carry out genome replication for ensuring faithful genome duplication by accurately synthesizing two identical copies of the parental DNA. Replisomes assemble at specific replication initiation sites known as origins, from where they initiate and carry out DNA synthesis. To ensure that chromosomes replicate once per cell cycle, overlapping regulatory mechanisms tightly control the initiation phase of replication and prevent unscheduled re-initiation events [1–3]. Failures in this control increase the risk of replication errors and double-stranded DNA breaks, compromising genetic stability and cell viability, and eventually causing diseases like cancer [4–7].

Since Schaechter's seminal studies in *Salmonella typhimurium* in 1958, it is now well established that replication is under metabolic control (this process is termed metabolic control of replication, MCR). In bacteria, this process impacts both the initiation and elongation phases of replication, resulting in a reproducible temporal compartmentalization of DNA synthesis in the cell cycle in a wide range of nutritional conditions and growth rates [8–12]. This system depends on the energy and precursors derived from nutrients rather than on the actual nature of the carbon and nitrogen sources, as *Escherichia coli* cells growing at the same rate in different media exhibit similar temporal replication patterns [9,11]. In lower eukaryotes, the compartmentalization of replication (S phase) also varies with growth and nutritional conditions [13,14]. Moreover, the cell cycle from yeast to human cells exhibits an autonomous metabolic/redox oscillation, and the entry and progression of the S phase occur at the most reductive phase of this cycle [15–17]. The metabolic oscillation also triggers global changes in the metabolome, including central carbon pathways [18–27]. Interestingly, metabolic oscillation may also be an intrinsic behaviour of bacteria as the cell cycle of *Caulobacter crescentus* is underpinned by robust metabolite fluctuations [28].

Soon after the Schaechter's seminal studies, MCR was assumed to be geared by a passive mechanism involving limiting factors. Initially, the pools of nucleotide precursors that increase with growth rate was proposed to directly determine the activity of replication enzymes and the rate of DNA synthesis. Although appealing, this hypothesis is challenged by data showing that artificial changes (balanced or imbalanced) in nucleotide pools increase replication errors, double-strand DNA breaks and ultimately genetic instability [4]. Subsequently, the ATP-bound form of the initiator DnaA that peaks near the time of initiation was proposed to be the limiting factor of MCR in bacteria [29]. However, this hypothesis is questioned by data showing that the timing of initiation poorly correlates to the amount of ATP-DnaA (or DnaA) per origin [30–35]. Hence, although ATP-DnaA is the motor of initiation, other factors are needed for proper temporalization of initiation with respect to nutritional conditions. Since the 2000s, MCR is thought to involve sophisticated signalling pathways that chemically sense the cellular metabolic state for temporalizing DNA synthesis in a broad range of nutritional conditions [36–40]. In this model, MCR continuously adjusts the size of precursor pools to the rate of DNA synthesis, preventing aberrant mutagenic feeding of replication enzymes.

**Figure 1.**
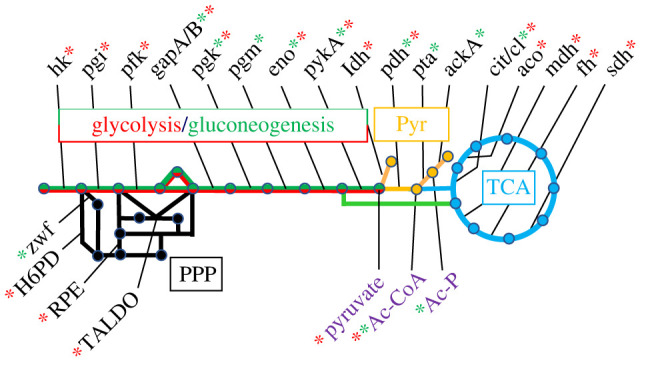
Schematic of central carbon metabolism and its main links to replication. Coloured lines and labels stand for glycolysis (red), gluconeogenesis (green), tricarboxylic acid cycle (TCA) (blue), pentose phosphate pathway (PPP) (dark), pyruvate metabolism (Pyr) (orange) and metabolites (violet). Key metabolic determinants (genes/enzymes and metabolites) connected to DNA replication in bacteria (green star) or in eukaryotes (red star) are listed using the following abbreviations: hk, hexokinase; pgi, glucose 6-phosphate isomerase; pfk, phosphofructokinase; gapA, glyceraldehyde 3-phosphate dehydrogenase, NAD-dependent; gapB, glyceraldehyde 3-phosphate dehydrogenase, NADP-dependent; pgk, phosphoglycerate kinase; pgm, phosphoglycerate mutase; eno, enolase; pykA, pyruvate kinase; ldh, lactate dehydrogenase; pta, phosphotransacetylase; ackA, acetate kinase; cit, citrate synthase; cl, ATP-citrate lyase; aco, aconitase; mdh, malate dehydrogenase; fh, fumarase; sdh, succinate dehydrogenase; zwf, glucose 6-phosphate dehydrogenase; H6PD, hexo-6-phosphate pyrophosphokinase; RPE, ribulose 5-phosphate epimerase; TALDO, transaldolase. Abbreviations of metabolites are as follows: Ac-CoA, acetyl-CoA; Ac-P, acetyl-phosphate.

The catabolic branch of metabolism, also called central carbon metabolism (CCM), encompasses approximately 40 ancestral, highly conserved enzymes organized in pathways (figure 1) [41,42]. It extracts from nutrients the precursors and energy needed for macromolecular synthesis and biomass production. By sensing the supply and demand in biosynthetic reactions, it also orchestrates metabolome homeostasis [43–45]. Because of its strategic position, CCM was proposed to play an important role in MCR. This hypothesis is supported by an ever-increasing body of data in both prokaryotic and eukaryotic cells linking CCM to replication (see below and figure 1 for a summary) and showing that CCM determinants often operate as signalling and sensor molecules ensuring moonlighting functions in non-metabolic activities [46]. Note that in addition to MCR, two systems are proposed to couple replication to growth. They involve molecules whose concentration reflects the metabolic state of the cell: the nucleotide analogues guanosine tetra- and penta-phosphate [47] (see however [34,48]) and reactive oxygen species [27,49–54].

## 2. CCM-replication links

In bacteria, replication initiation involves assembly of multiple copies of the initiator protein DnaA at the origin (*oriC*) to form a nucleoprotein complex that triggers origin unwinding, replicative helicase loading, replisome assembly and replication firing [55,56]. Two CCM metabolites were suggested to modulate this replication step in *E. coli*: acetyl-CoA and acetyl-phosphate. These metabolites acetylate DnaA through an acetyltransferase (YfiQ) or non-enzymatically, respectively, collectively inhibiting ATP and ADP binding to DnaA and impeding formation of the active DnaA-origin nucleoprotein complex [57]. DnaA acetylation is removed by the deacetylase CobB. Although DnaA acetylation/deacetylation is documented *in vivo* and *in vitro*, its exact contribution to initiation regulation in steady-state growing cells remains uncertain, as DnaA acetylation is so far only known to peak at the stationary phase compared to the exponential growth phase. Interestingly, another study links the acetylation potential to DnaA activity. In that work, a general decrease in protein acetylation induced by CCM mutations depleting the pools of acetyl-CoA and acetyl-phosphate or by deleting the major acetyltransferase *yfiQ* was found to suppress initiation defects of a DnaA mutant (*dna46*) [58]. Despite this, the authors were unable to conclude on the role of DnaA acetylation in initiation regulation in exponentially growing cells, as suppression still occurs in conditions of general increase in acetylation (in *cobB* knockout cells). Instead, they proposed various scenarios where initiation is modulated by other metabolic determinants impacting formation of the DnaA-origin nucleoprotein complex. One such scenario may involve cyclic AMP which interacts *in vitro* with DnaA and promotes the production of the active DnaA-ATP form and its binding to the replication origin [59].

In addition to metabolites, key CCM enzymes are also involved in replication. In *Bacillus subtilis*, several catabolic enzymes are linked to three replication proteins essential for the initiation and elongation phases of replication: the DnaC helicase, the DnaG primase and the lagging strand DNA polymerase DnaE. These proteins are loaded early at the origin during initiation, and may form a ternary complex in the replisome to carry out DNA melting, RNA priming and lagging strand synthesis during DNA polymerization [60,61]. The first group of CCM enzymes important for replication are subunits of the pyruvate dehydrogenase PDH and related enzymes. They interact with DnaC, DnaG and the origin region and inhibit initiation [62–65]. Recently, our laboratories reported that the pyruvate kinase PykA modulates *in vitro* the activities of DnaC, DnaG and DnaE, and that the catalytic (CAT) domain of PykA interacts with DnaE when the polymerase is bound to primed DNA templates [66,67]. *In vivo*, PykA impacts replication in two ways: the CAT domain stimulates elongation while the C-terminal PEPut (phosphoenolpyruvate utilizer) domain inhibits initiation. Interestingly, these phenotypes are independent from PykA catalytic activity and occur in conditions where the enzyme is dispensable for growth (e.g. in a gluconeogenic medium) and does not significantly contribute to the metabolome. Therefore, the replication defects in PykA mutants are a direct consequence of amino acid residue changes rather than an indirect consequence of changes in cellular metabolism. It was thus proposed that PykA typifies a new family of replication control factors that gears MCR [66].

Genetic studies have been carried out to investigate the CCM-replication links more comprehensively. In *B. subtilis*, these studies uncovered a toolbox of nutrient-dependent activated CCM-replication links important for replication temporalization. This toolbox comprises on one side, CCM reactions ensuring the 3C part of glycolysis and reactions of the downstream pyruvate metabolism, and on the other side, the replication enzymes DnaC, DnaG and DnaE. Mutations in this CCM area suppress replication defects in *dnaC*, *dnaG* and *dnaE* (but not in *dnaI*, *dnaD*, *polC*, *dnaX* and *dnaN*) conditional mutants through a process involving conformational changes in replication enzymes [68]. These mutations also alter initiation and elongation in a medium-dependent manner and disturb the metabolic control of replication [34,69]. Similar links were described in *E. coli* [70–72] and possibly *C. crescentus* [73].

In eukaryotes, CCM determinants (proteins and metabolites) found in the nucleus [74–76] often exert moonlighting replication functions. For instance, the timing of origin firing depends on histone acetylation promoted by an increase in nuclear acetyl-CoA. This increase is geared by the redox metabolic cycle in yeast, and by nuclear forms of the ATP-citrate lyase and PDH complexes in mammalian cells [77–80]. In addition, the nuclear glyceraldehyde-3-phosphate dehydrogenase (GAPDH) and lactate dehydrogenase (LDH) in human cells are integral parts of the transcription cofactor OCA-S [81,82]. In response to the local NAD^+^/NADH redox status, these metabolic enzymes control the association of OCA-S with the transcription factor Oct-1 for regulating histone H2B expression and S phase progression. Moreover, core histone gene expression and S phase entry are impeded by exogenous pyruvate [83] and chromatin and chromatin factors are important for origin selection, fork rate optimization and lagging strand priming [84–86]. Collectively, these data suggest that CCM metabolites and enzymes playing fundamental role in histone biology and gene expression [75,87] also ensure important MCR functions. However, eukaryote MCR may also operate through histone-independent pathways. Indeed, the phosphoglycerate kinase PGK interacts with the protein kinase CDC7 to stimulate replication initiation by enhancing the CDC7-mediated activation of the replicative MCM helicase in human cells [88]. Also, mammalian LDH, PGK and GAPDH modulate the activity of the replicative polymerases Pol*α*, Pol*ε* and Pol*δ in vitro* [89–91]. We may also note here that impaired expression of CCM genes delays the entry of human fibroblasts into the S phase or decreases the number of cells in this phase [92–94]. Collectively, these data show that CCM impacts both the initiation and elongation phases of replication from bacteria to humans.

## 3. Basic principles of MCR

**Figure 2.**
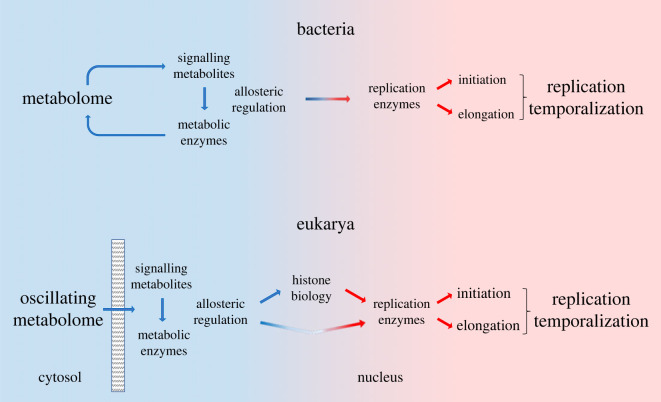
Basic principles of the metabolic control of replication. (*a*) Model for bacteria. CCM signalling metabolites allosterically regulate CCM enzymes for adjusting the metabolome to the energy afforded by available nutrients. The information contained in signalling metabolites and metabolic enzyme conformers is then conveyed to replication enzymes for regulating initiation and elongation and temporalizing replication in the cell cycle in a broad range of nutritional conditions. The transmission of information may involve physical interactions between CCM determinants and replication enzymes. (*b*) Model for eukaryotes. The cellular metabolism oscillates spontaneously and regulates cell cycle progression. Nuclear signalling metabolites and CCM enzymes sense and convey this information for modulating accordingly replication enzyme activity and replication temporalization through direct (physical interactions between CCM determinants and replication proteins) or indirect (through histone biology) pathways. The dashed box represents the nuclear membrane.

Here, we propose that MCR is orchestrated by an active process organized as a toolbox of overlapping mechanisms differently activated in response to nutritional stimuli. In this toolbox, signalling metabolites and cognate CCM proteins convey metabolic information to enzymes of the initiation and elongation replication machineries for coupling replication temporalization to metabolism (figure 2). The transmission of metabolic information is thought to involve sophisticated (indirect) mechanisms (e.g. MCM helicase activation by PGK via CDC7 [88]; initiation firing by acetyl-CoA via histone acetylation [77–80]), although simpler (direct) mechanisms could also operate (e.g. cAMP binding to DnaA [59]). Interestingly, MCR appears to have common roots with metabolism regulation, as the CCM determinants committed to replication often continuously signal and sense metabolic fluctuations for achieving metabolome homeostasis. Hence, our MCR model implies that allosteric regulation of CCM enzymes by signalling metabolites coordinates simultaneously metabolome homeostasis and replication temporalization. The numerous CCM-replication links described above potentially highlight the diversity of these coordinating systems and similar mechanisms may be at play for integrating many cellular behaviours to metabolism [46]. Molecular insights in the *B. subtilis* PykA-driven MCR are presented below.

## 4. PykA-driven MCR in *B. subtilis*

### 4.1. PykA allosteric regulation

**Figure 3.**
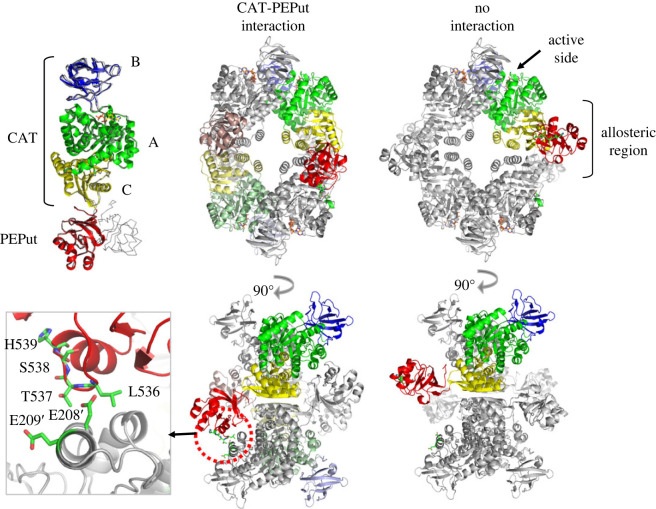
AlphaFold2 analysis of *B. subtilis* PykA conformers. Top left panel: AlphaFold2 predicted structure (ranked 0) of the PykA monomer coloured to highlight the A, B and C domains of the catalytic region (CAT) and PEPut. A monomer with a lower ranking (19) is superposed as a cartoon onto the coloured structure. Both structures are highly similar, differing mostly by an alternative PEPut position. Right panel: tetrameric structures built from the ranked 0 (leftward configuration) and ranked 19 (rightward configuration) monomer predictions (two views of each tetramer are provided). They mostly differ in the PEPut position. Inlet: Close-up view of the PEPut-A' region of the left tetrameric structure suggesting a H-bound interaction between L536 (PEPut) and E208′ (A').

**Figure 4.**
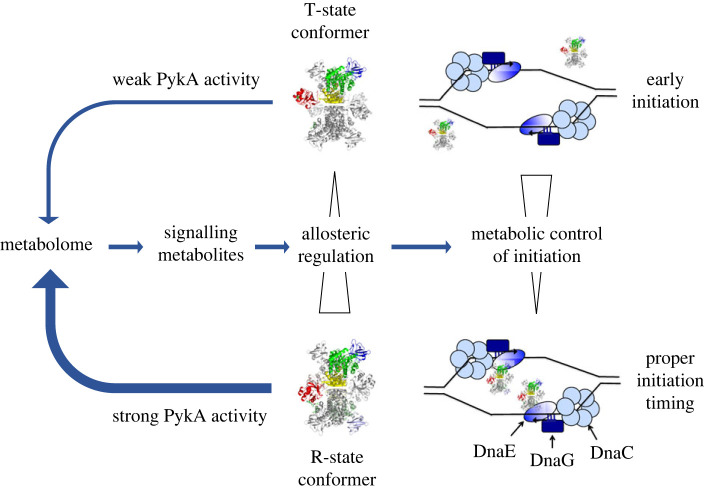
Roles of PykA conformers in metabolome homeostasis and replication compartmentalization. Left panel: CCM signalling metabolites allosterically regulate the ratio between the active (R-state) versus inactive (T-state) PykA conformers for achieving metabolome homeostasis. Right panel: the R-state PykA conformer is recruited by DnaE bound to primed DNA at initiation sites for ensuring a wild-type initiation timing. The T-state-conformer does not interact with DnaE at initiation sites allowing early initiation firing.

Pyruvate kinases are ancestral, highly conserved homotetramers that carry out the last reaction of glycolysis and anaplerotic activities in gluconeogenic conditions. Almost all pyruvate kinases are homotropically activated by the substrate phosphoenolpyruvate and allosterically regulated by heterotrophic effectors [95]. The mechanism of allosteric regulation involves conformational changes within each subunit and between neighbouring subunits for stabilizing the protein in the metabolically active R-state conformation [95,96]. As mentioned above, the *B. subtilis* PykA protein comprises a CAT (catalytic) domain containing the substrate and effector binding sites, and a PEPut domain. This latter domain is highly similar to the PEP utilizer domain of pyruvate orthophosphate dikinase and other metabolic enzymes [97]. In these enzymes, the PEP utilizer moves around a flexible arm [98,99] and is phosphorylated at a conserved TSH motif at the expense of phosphoenolpyruvate and ATP to drive sugar imports and catalytic or regulatory activities [98,100–106]. The PEPut domain of PykA is not required for the catalytic activity of the enzyme [67,107]. However, it interacts with CAT via a hydrogen bond between E208 (CAT) and L536 (PEPut), as shown in a structural study of the highly homologous (72.5% identity) *Geobacillus stearothermophilus* PykA [108]. In *B. subtilis*, this interaction seems to stimulate PykA activity and to be negatively regulated by phosphorylation of the T residue of the TSH motif which maps immediately downstream of the interacting L536 amino acid (residues 537–539) [66,67]. An AlphaFold2 analysis predicts two *B. subtilis* PykA monomer structures with a highly similar spatial CAT domain and alternative PEPut positions (figure 3, top left panel) [109,110], indicating that PEPut is carried by a flexible arm, as in other PEP utilizer containing metabolic enzymes [98,99]. The tetrameric structures built from these monomeric predictions also mostly differ in the PEPut position (figure 3, middle and right panels): this peptide faces the A' domain of CAT of another subunit in the left configuration while it is directed outward of CAT in the right configuration. A close-up view (figure 3, inset) of the PEPut-A' region of the left structure suggests a H-bond interaction between L536 (PEPut) and E208′ (A'), as expected from crystallography studies [108]. Hence, the AlphaFold2 study predicted two conformers of *B. subtilis* PykA tetramers in which PEPut interacts (left configuration) or not (right configuration) with CAT. These configurations are proposed to be related to the classical R- and T-state of PykA conformers, respectively [96]. The proximity of T537 with the E208′-L536 H-bound (figure 3, inset) suggests that T537 phosphorylation inhibits the CAT-PEPut interaction, R-state conformer formation and PykA activity by bringing positive charges at the CAT-PEPut interface. We infer from these results that the CAT-PEPut interaction assists the heterotrophic effectors (AMP and ribose 5-phosphate [111]) in stabilizing the active R-state conformer and that this stimulation is negatively regulated by T537 phosphorylation [66,67,96]. Hence, PykA is a metabolic sensor that resides in individual cells in different conformations at levels depending on ligand (PEP, AMP and ribose 5-phosphate) concentrations and kinase activities that control PEPut phosphorylation and the CAT-PEPut interaction to provide the active R-state conformer at a concentration sufficient for biosynthetic needs and metabolome homeostasis (figure 4, left panel). We foresee that the pattern of PykA conformers is used by cells for dynamically characterizing nutritional conditions.

### 4.2. PykA-driven MCR

In addition to being a key regulator of PykA catalytic activity and metabolome homeostasis, PEPut operates as a negative regulator of replication initiation in gluconeogenic growth conditions [66]. Interestingly, this function strongly depends on determinants involved in PykA conformers regulation, namely the CAT-PEPut interaction and T537 phosphorylation. When these determinants favour formation of the R-state conformer (i.e. when PEPut efficiently interacts with CAT), a wild-type initiation activity is found while cells over-initiate in conditions favouring the accumulation of the T-state conformer (i.e. when the CAT-PEPut interaction is impeded) (figure 4, right panel). In addition to these distinct effects of PykA conformers on initiation, it was shown that (i) PykA physically interacts with the polymerase DnaE via its CAT domain when the replication enzyme is bound to primed DNA [67], (ii) PEPut modulates the strength of this interaction and its effect on the DnaE polymerase activity [66,67], and (iii) DnaE is early recruited at replication origins during initiation [61]. We thus suggest from these data that the MCR initiation depends on the effect of PykA conformers on DnaE activity during initiation. Although different mechanisms can be envisioned at this stage of our knowledge, we tentatively propose a model in which only the R-state conformer interacts with DnaE at initiation sites for reducing the DnaE polymerase activity and preventing early initiation (figure 4, right panel). In an alternative model (not shown), both conformers are recruited at initiation sites by DnaE. However, while the T-state conformer dramatically increases DnaE activity and causes early initiation, the R-state conformer allows a proper polymerase activity and initiation timing. Together, these results suggest that the signalling system gearing the balance between the R- and T-state PykA conformers controls simultaneously and dynamically PykA activity, metabolome homeostasis and MCR initiation. However, although cells allosterically regulate PykA catalytic activity in a broad range of nutritional conditions, the PEPut-driven initiation function of PykA is active in gluconeogenic conditions but inactive in glycolytic media [66]. This suggests that the initiation function of PykA conformers depends on the polarity of the carbon flux travelling CCM and that cells temporalize initiation through different mechanisms in glycolytic and gluconeogenic conditions.

As mentioned above, CAT stimulates DNA elongation in gluconeogenic growth conditions and this moonlighting activity depends on substrates (ADP and phosphoenolpyruvate) binding but not on PykA catalytic activity [66]. Moreover, CAT physically interacts with DnaE when the polymerase is bound to primed DNA and DnaE is frequently recruited to and released from replication forks to initiate DNA synthesis from the RNA primers produced during lagging strand synthesis [60,112–114]. Together, these results suggest that PykA is repeatedly recruited by DnaE in the replisome, allowing a highly dynamic transmission of the metabolic information contained in PykA conformers to replication forks. By modulating DnaE activity and fork speed, this system is proposed to gear the MCR elongation.

## 5. Conclusion and perspectives

In both prokaryotic and eukaryotic cells, CCM does more than simply producing precursors and energy for anabolic reactions. It also drives fundamental biological processes. To do so, many CCM metabolites and enzymes operate as signalling and sensor molecules that not only carry out metabolome homeostasis but also ensure moonlighting functions in non-metabolic activities [46]. DNA synthesis is demanding in energy and precursors. It must also be completed once initiated to prevent DNA damage that risk cell survival and health. It is therefore not surprising that DNA replication is under a metabolic control. The ever-increasing number of reports since the 1960s describing the impact of nutritional conditions on DNA replication temporalization and tight links between CCM and replication from bacteria to humans highlights the importance of such a control in all living organisms. However, despite significant efforts, the molecular mechanism and biological function of MCR remain largely unknown. This is likely due to the fact that this control involves a toolbox of complex and overlapping processes differently activated in response to nutritional stimuli. Because of their genetic tractability, relative cellular simplicity and robust cell cycle response to nutrient fluctuations, bacteria are attractive models for unraveling the mechanism and function of MCR. The recent discovery that side chain mutations in the *B. subtilis* PykA dramatically impact replication temporalization independently of their effect on PykA catalytic activity and cellular metabolome validates this notion and paves the way for further studies in this bacterium as well as in distantly related organisms. Insights from this and other organisms suggest that (i) CCM elements that signal and sense the cellular metabolic state for ensuring metabolome homeostasis are key players in MCR, and that (ii) the metabolic information contained in signalling molecules is conveyed to the replication machinery through protein-protein interactions physically connecting metabolic and replication enzymes and/or by inducing conformational changes in histone proteins via post-translational modifications. We foresee that the CCM-replication links discussed above highlight new PykA-like multifunctional regulators from bacteria to humans. Finally, it is interesting to note here that the MCR model proposed here suggests that DNA replication depends on a few key signalling metabolites in addition to the well-characterized classical replication control functions [1–3].

Surprisingly, *B. subtilis* mutants of MCR suffer from profound initiation and elongation defects, causing dramatic changes in replication compartmentalization and cell cycle [66]. As these phenotypes occur in cells fully proficient in ‘classical' replication control functions [1–3], these results show that MCR plays an important role in DNA synthesis and that the control geared by ‘classical' replication functions is not sufficient to properly time DNA synthesis in a broad range of nutritional conditions. Regulation of DNA replication is of prime importance for faithful propagation of the genetic material to daughter cells. Failures in this control cause chromosomal lesions (double-strand DNA breaks, nucleotide misincorporations) with a high mutagenic potential that increases the risk of genetic diseases like cancer [4–7]. Towards the beginning of the twentieth century, it was shown that cancer cells exhibit chromosomal abnormalities, elevated glucose uptake and accelerated glycolysis flux to lactate even in the presence of sufficient oxygen (Warburg effect). We know now that genome instability and altered metabolism are two hallmarks of cancer [115]. Genome instability has been investigated continuously since its discovery leading to the proposal that it is due to DNA replication stresses caused by deregulation of initiation and fork speed. A large core of data shows that these replication abnormalities are induced by tumorigenesis drivers like oncogenes, tumour suppressors and/or mutator genes [116–119]. On the other side, altered metabolism has been rejuvenated as a research area only recently and since then, it was shown that the Warburg effect is a crucial component of malignancy that satisfies bioenergetic and biosynthetic demands, mitigates oxidative stress and contributes to cell transformation and metastasis [120,121]. Nowadays, cancer is thought to originate from mutations caused by replication stresses, and other hallmarks of cancer, including the altered metabolism, are thought to be a consequence of these mutations rather than the cause [116,122,123]. However, recent studies have shown that altered metabolism impacts genetic stability. Indeed, mutations in CCM enzymes and changes in the level of CCM metabolites were shown to directly cause mutagenic DNA damage, impede DNA repair and trigger nucleotide imbalance [124–126]. Moreover, CCM mutations disrupting MCR may cause replication phenotypes known to increase genetic instability, and alteration of the synchrony between the reductive stage of the metabolic cycle and the S phase in yeast increases the rate of spontaneous mutagenesis [66,127]. Collectively, these data suggest that CCM changes underpinning the Warburg effect may be an additional root cause of genetic instability and cancer initiation. It is of interest to note here that the past two decades have shown that many metabolites produced by the gut microbiome of mammals enter the bloodstream, impacting the blood metabolome and the chemical environment of tissues and host cells [128–130]. In humans, this transport is a major determinant of blood metabolite variability and human health [131–135]. Moreover, the cross-talk between bacterial metabolites, histone biology and cancer highlights the links between altered microbiomes (dysbiosis) and intracellular pools of chemicals involved in DNA damage, DNA repair and potentially MCR [136–138]. Finally, it is becoming increasingly clear that metabolism plays an active role through moonlighting functions in cellular processes as diverse as transcription, posttranslational modifications, cell division, apoptosis, pathogenicity, innate immunity and development [22,36,74–76,139–147]. Therefore, CCM may provide a toolbox of links connecting determinants gearing metabolome homeostasis to a wide range of cellular processes in addition to DNA replication. Future efforts should focus on revealing the extent and mechanisms by which these determinants may actively drive and tune main cellular functions.

## Data Availability

This article has no additional data.

## References

[1] Sclafani RA, Holzen TM. 2007 Cell cycle regulation of DNA replication. Annu. Rev. Genet. **41**, 237-280. (10.1146/annurev.genet.41.110306.130308) 17630848 PMC2292467

[2] Skarstad K, Katayama T. 2013 Regulating DNA replication in bacteria. Cold Spring Harb. Perspect. Biol. **5**, a012922. (10.1101/cshperspect.a012922) 23471435 PMC3683904

[3] Jameson K, Wilkinson A. 2017 Control of initiation of DNA replication in *Bacillus subtilis* and *Escherichia coli*. Genes **8**, 22. (10.3390/genes8010022) 28075389 PMC5295017

[4] Mathews CK. 2015 Deoxyribonucleotide metabolism, mutagenesis and cancer. Nat. Rev. Cancer. **15**, 528-539. (10.1038/nrc3981) 26299592

[5] Tubbs A, Nussenzweig A. 2017 Endogenous DNA damage as a source of genomic instability in cancer. Cell **168**, 644-656. (10.1016/j.cell.2017.01.002) 28187286 PMC6591730

[6] Rodgers K, McVey M. 2016 Error-prone repair of DNA double-strand breaks: error-prone repair of DNA DSBs. J. Cell Physiol. **231**, 15-24. (10.1002/jcp.25053) 26033759 PMC4586358

[7] Saxena S, Zou L. 2022 Hallmarks of DNA replication stress. Mol. Cell **82**, 2298-2314. (10.1016/j.molcel.2022.05.004) 35714587 PMC9219557

[8] Schaechter M, MaalOe O, Kjeldgaard NO. 1958 Dependency on medium and temperature of cell size and chemical composition during balanced growth of *Salmonella typhimurium*. J. Gen. Microbiol. **19**, 592-606. (10.1099/00221287-19-3-592) 13611202

[9] Helmstetter CE. 1996 Timing of synthetic activities in the cell cycle. In Escherichia coli and salmonella: cellular and molecular biology (eds FC Neidhardt, R Curtis III, JL Ingraham, ECC Lin), pp. 1627-1639. Washington, DC: ASM Press.

[10] Sharpe ME, Hauser PM, Sharpe RG, Errington J. 1998 *Bacillus subtilis* cell cycle as studied by fluorescence microscopy: constancy of cell length at initiation of DNA replication and evidence for active nucleoid partitioning. J. Bacteriol. **180**, 547-555. (10.1128/JB.180.3.547-555.1998) 9457856 PMC106920

[11] Michelsen O, Teixeira de Mattos MJ, Jensen PR, Hansen FG. 2003 Precise determinations of C and D periods by flow cytometry in *Escherichia coli* K-12 and B/r. Microbiology **149**, 1001-1110. (10.1099/mic.0.26058-0) 12686642

[12] Stokke C, Flåtten I, Skarstad K. 2012 An easy-to-use simulation program demonstrates variations in bacterial cell cycle parameters depending on medium and temperature. PLoS ONE **7**, e30981. (10.1371/journal.pone.0030981) 22348034 PMC3278402

[13] Rivin CJ, Fangman WL. 1980 Cell cycle phase expansion in nitrogen-limited cultures of *Saccharomyces cerevisiae*. J. Cell Biol. **85**, 96-107. (10.1083/jcb.85.1.96) 6988443 PMC2110600

[14] Carlson C, Grallert B, Stokke BE. 1999 Regulation of the start of DNA replication in *Schizosaccharomyces pombe*. J. Cell Sci **112**, 939-946. (10.1242/jcs.112.6.939) 10036243

[15] Klevecz RR, Bolen J, Forrest G, Murray DB. 2004 A genomewide oscillation in transcription gates DNA replication and cell cycle. Proc. Natl Acad. Sci. USA **101**, 1200-1205. (10.1073/pnas.0306490101) 14734811 PMC337030

[16] Tu BP, Kudlicki A, Rowicka M, McKnight SL. 2005 Logic of the yeast metabolic cycle: temporal compartmentalization of cellular processes. Science **310**, 1152-1158. (10.1126/science.1120499) 16254148

[17] Burnetti AJ, Aydin M, Buchler NE. 2016 Cell cycle start is coupled to entry into the yeast metabolic cycle across diverse strains and growth rates. Mol. Biol. Cell **27**, 64-74. 26538026 10.1091/mbc.E15-07-0454 PMC4694762

[18] Tu BP et al. 2007 Cyclic changes in metabolic state during the life of a yeast cell. Proc. Natl Acad. Sci. USA **104**, 16 886-16 891. (10.1073/pnas.0708365104) PMC2040445 17940006

[19] Vizán P, Alcarraz-Vizán G, Díaz-Moralli S, Solovjeva ON, Frederiks WM, Cascante M. 2009 Modulation of pentose phosphate pathway during cell cycle progression in human colon adenocarcinoma cell line HT29. Int. J. Cancer **124**, 2789-2796. (10.1002/ijc.24262) 19253370

[20] Ewald JC, Kuehne A, Zamboni N, Skotheim JM. 2016 The yeast cyclin-dependent kinase routes carbon fluxes to fuel cell cycle progression. Mol. Cell **62**, 532-545. (10.1016/j.molcel.2016.02.017) 27203178 PMC4875507

[21] Zhao G, Chen Y, Carey L, Futcher B. 2016 Cyclin-dependent kinase co-ordinates carbohydrate metabolism and cell cycle in *S. cerevisiae**.* Mol. Cell **62**, 546-557. (10.1016/j.molcel.2016.04.026) 27203179 PMC4905568

[22] Papagiannakis A, Niebel B, Wit EC, Heinemann M. 2017 Autonomous metabolic oscillations robustly gate the early and late cell cycle. Mol. Cell **65**, 285-295. (10.1016/j.molcel.2016.11.018) 27989441

[23] Ahn E, Kumar P, Mukha D, Tzur A, Shlomi T. 2017 Temporal fluxomics reveals oscillations in TCA cycle flux throughout the mammalian cell cycle. Mol. Syst. Biol. **13**, 953. (10.15252/msb.20177763) 29109155 PMC5731346

[24] Roci I, Watrous JD, Lagerborg KA, Jain M, Nilsson R. 2020 Mapping metabolic oscillations during cell cycle progression. Cell Cycle **19**, 2676-2684. (10.1080/15384101.2020.1825203) 33016215 PMC7644150

[25] Liu J et al. 2021 Skp2 dictates cell cycle-dependent metabolic oscillation between glycolysis and TCA cycle. Cell Res. **31**, 80-93. (10.1038/s41422-020-0372-z) 32669607 PMC7852548

[26] Campbell K, Westholm J, Kasvandik S, Di Bartolomeo F, Mormino M, Nielsen J. 2020 Building blocks are synthesized on demand during the yeast cell cycle. Proc. Natl Acad. Sci. USA **117**, 7575-7583. (10.1073/pnas.1919535117) 32213592 PMC7149230

[27] Kirova DG et al. 2022 A ROS-dependent mechanism promotes CDK2 phosphorylation to drive progression through S phase. Dev. Cell. **57**, 1712-1727. (10.1016/j.devcel.2022.06.008) 35809563 PMC9616724

[28] Hartl J et al. 2020 Untargeted metabolomics links glutathione to bacterial cell cycle progression. Nat. Metab. **2**, 153-166. (10.1038/s42255-019-0166-0) 32090198 PMC7035108

[29] Kurokawa K. 1999 Replication cycle-coordinated change of the adenine nucleotide-bound forms of DnaA protein in *Escherichia coli*. EMBO J. **18**, 6642-6652. (10.1093/emboj/18.23.6642) 10581238 PMC1171727

[30] Koppes LJH. 1987 OriC plasmids do not affect timing of chromosome replication in *Escherichia coli* K12. Mol. Gen. Genet. **209**, 188-192. (10.1007/BF00329857) 3312956

[31] Torheim NK, Boye E, Løbner-Olesen A, Stokke T, Skarstad K. 2002 The *Escherichia coli* SeqA protein destabilizes mutant DnaA204 protein: DnaA protein degradation. Mol. Microbiol. **37**, 629-638. (10.1046/j.1365-2958.2000.02031.x) 10931356

[32] Flåtten IM, Skarstad K. 2009 DnaA protein interacts with RNA polymerase and partially protects it from the effect of rifampicin. Mol. Microbiol. **71**, 1018-1030. (10.1111/j.1365-2958.2008.06585.x) 19170875

[33] Charbon G et al. 2011 Suppressors of DnaAATP imposed overinitiation in *Escherichia coli*: suppressors of Hda deficiency in *E. coli*. Mol. Microbiol. **79**, 914-928. (10.1111/j.1365-2958.2010.07493.x) 21299647

[34] Murray H, Koh A. 2014 Multiple regulatory systems coordinate DNA replication with cell growth in *Bacillus subtilis*. PLoS Genet. **10**, e1004731. (10.1371/journal.pgen.1004731) 25340815 PMC4207641

[35] Flåtten I, Fossum-Raunehaug S, Taipale R, Martinsen S, Skarstad K. 2015 The dnaA protein is not the limiting factor for initiation of replication in *Escherichia coli*. Burkholder WF, editor. PLOS Genet. **11**, e1005276. (10.1371/journal.pgen.1005276) 26047361 PMC4457925

[36] Wang JD, Levin PA. 2009 Metabolism, cell growth and the bacterial cell cycle. Nat. Rev. Microbiol. **7**, 822-827. (10.1038/nrmicro2202) 19806155 PMC2887316

[37] Buchakjian MR, Kornbluth S. 2010 The engine driving the ship: metabolic steering of cell proliferation and death. Nat. Rev. Mol. Cell Biol. **11**, 715-727. (10.1038/nrm2972) 20861880

[38] Barańska S et al. 2013 Replicating DNA by cell factories: roles of central carbon metabolism and transcription in the control of DNA replication in microbes, and implications for understanding this process in human cells. Microb. Cell Factories. **12**, 55. (10.1186/1475-2859-12-55) PMC3698200 23714207

[39] Ewald JC. 2023 How yeast coordinates metabolism, growth and division. Curr. Opin. Microbiol. **45**, 1-7. (10.1016/j.mib.2017.12.012) 29334655

[40] He H, Lee MC, Zheng LL, Zheng L, Luo Y. 2013 Integration of the metabolic/redox state, histone gene switching, DNA replication and S-phase progression by moonlighting metabolic enzymes. Biosci. Rep. **33**, e00018. (10.1042/BSR20120059) 23134369 PMC3561917

[41] Canback B, Andersson SGE, Kurland CG. 2002 The global phylogeny of glycolytic enzymes. Proc. Natl Acad. Sci. USA **99**, 6097-6102. (10.1073/pnas.082112499) 11983902 PMC122908

[42] Zhao W et al. 2022 Proteome-wide 3D structure prediction provides insights into the ancestral metabolism of ancient archaea and bacteria. Nat. Commun. **13**, 7861. (10.1038/s41467-022-35523-8) 36543797 PMC9772386

[43] Fujita Y. 2009 Carbon catabolite control of the metabolic network in *Bacillus subtilis*. Biosci. Biotechnol. Biochem. **73**, 245-259. (10.1271/bbb.80479) 19202299

[44] Chubukov V, Gerosa L, Kochanowski K, Sauer U. 2014 Coordination of microbial metabolism. Nat. Rev. Microbiol. **12**, 327-340. (10.1038/nrmicro3238) 24658329

[45] Liu GY, Sabatini DM. 2020 mTOR at the nexus of nutrition, growth, ageing and disease. Nat. Rev. Mol. Cell Biol. **21**, 183-203. (10.1038/s41580-019-0199-y) 31937935 PMC7102936

[46] Baker SA, Rutter J. 2023 Metabolites as signalling molecules. Nat. Rev. Mol. Cell Biol. **24**, 355-374. (10.1038/s41580-022-00572-w) 36635456

[47] Beaufay F, Coppine J, Hallez R. 2021 When the metabolism meets the cell cycle in bacteria. Curr. Opin. Microbiol. **60**, 104-113. (10.1016/j.mib.2021.02.006) 33677348

[48] Hernandez VJ, Bremer H. 1993 Characterization of RNA and DNA synthesis in *Escherichia coli* strains devoid of ppGpp*. J. Biol. Chem. **268**, 10 851-10 862. (10.1016/S0021-9258(18)82063-4) 7684368

[49] McBride AA, Klausner RD, Howley PM. 1992 Conserved cysteine residue in the DNA-binding domain of the bovine papillomavirus type 1 E2 protein confers redox regulation of the DNA-binding activity in vitro. Proc. Natl Acad. Sci. USA **89**, 7531-7535. (10.1073/pnas.89.16.7531) 1323841 PMC49744

[50] Sanders CM, Sizov D, Seavers PR, Ortiz-Lombardia M, Antson AA. 2007 Transcription activator structure reveals redox control of a replication initiation reaction. Nucleic Acids Res. **35**, 3504-3515. (10.1093/nar/gkm166) 17478495 PMC1904295

[51] Sela D, Yaffe N, Shlomai J. 2008 Enzymatic mechanism controls redox-mediated protein-DNA interactions at the replication origin of kinetoplast DNA minicircles. J. Biol. Chem. **283**, 32 034-32 044. (10.1074/jbc.M804417200) 18799461

[52] O'Brien E et al. 2017 The [4Fe4S] cluster of human DNA primase functions as a redox switch using DNA charge transport. Science **355**, eaag1789. (10.1126/science.aag1789) 28232525 PMC5338353

[53] Bartels PL, Stodola JL, Burgers PMJ, Barton JK. 2017 A redox role for the [4Fe4S] cluster of yeast DNA polymerase *δ*. J. Am. Chem. Soc. **139**, 18 339-18 348. (10.1021/jacs.7b10284) PMC5881389 29166001

[54] O'Brien E, Salay LE, Epum EA, Friedman KL, Chazin WJ, Barton JK. 2018 Yeast require redox switching in DNA primase. Proc. Natl Acad. Sci. USA **115**, 13 186-13 191. (10.1073/pnas.1810715115) PMC6310810 30541886

[55] Leonard AC, Rao P, Kadam RP, Grimwade JE. 2019 Changing perspectives on the role of DnaA-ATP in Orisome function and timing regulation. Front. Microbiol. **10**, 2009. (10.3389/fmicb.2019.02009) 31555240 PMC6727663

[56] Hansen FG, Atlung T. 2018 The DnaA tale. Front. Microbiol. **9**, 319. (10.3389/fmicb.2018.00319) 29541066 PMC5835720

[57] Zhang Q et al. 2016 Reversible lysine acetylation is involved in DNA replication initiation by regulating activities of initiator DnaA in *Escherichia coli*. Sci Rep. **6**, 30 837. (10.1038/srep30837) PMC4971506 27484197

[58] Tymecka-Mulik J et al. 2017 Suppression of the *Escherichia coli* dnaA46 mutation by changes in the activities of the pyruvate-acetate node links DNA replication regulation to central carbon metabolism. Korolev S, editor. PLoS ONE **12**, e0176050. (10.1371/journal.pone.0176050) 28448512 PMC5407757

[59] Hughes P, Landoulsi A, Kohiyama M. 1988 A novel role for cAMP in the control of the activity of the *E. coli* chromosome replication initiator protein, DnaA. Cell **55**, 343-350. (10.1016/0092-8674(88)90057-8) 2844416

[60] Rannou O et al. 2013 Functional interplay of DnaE polymerase, DnaG primase and DnaC helicase within a ternary complex, and primase to polymerase hand-off during lagging strand DNA replication in *Bacillus subtilis*. Nucleic Acids Res. **41**, 5303-5320. (10.1093/nar/gkt207) 23563155 PMC3664799

[61] Paschalis V, Le Chatelier E, Green M, Képès F, Soultanas P, Janniere L. 2017 Interactions of the *Bacillus subtilis* DnaE polymerase with replisomal proteins modulate its activity and fidelity. Open Biol. **7**, 170146. (10.1098/rsob.170146) 28878042 PMC5627055

[62] Laffan J, Firshein W. 1987 Membrane protein binding to the origin region of *Bacillus subtilis*. J. Bacteriol. **169**, 4135-4140. (10.1128/jb.169.9.4135-4140.1987) 3114234 PMC213720

[63] Laffan JJ, Firshein W. 1988 Origin-specific DNA-binding membrane-associated protein may be involved in repression of initiation of DNA replication in *Bacillus subtilis*. Proc. Natl Acad. Sci. USA **85**, 7452-7456. (10.1073/pnas.85.20.7452) 3140241 PMC282209

[64] Stein A, Firshein W. 2000 Probable Identification of a membrane-associated repressor of *Bacillus subtilis* DNA Replication as the e2 subunit of the pyruvate dehydrogenase complex. J. Bacteriol. **182**, 2119-2124. (10.1128/JB.182.8.2119-2124.2000) 10735853 PMC111259

[65] Noirot-Gros MF et al. 2002 An expanded view of bacterial DNA replication. Proc. Natl Acad. Sci. USA **99**, 8342-8347. (10.1073/pnas.122040799) 12060778 PMC123069

[66] Horemans S et al. 2022 Pyruvate kinase, a metabolic sensor powering glycolysis, drives the metabolic control of DNA replication. BMC Biol. **20**, 87. (10.1186/s12915-022-01278-3) 35418203 PMC9009071

[67] Holland A, Pitoulias M, Soultanas P, Janniere L. 2023 The replicative DnaE polymerase of bacillus subtilis recruits the glycolytic pyruvate kinase (PykA) when bound to primed DNA templates. Life **13**, 965. (10.3390/life13040965) 37109494 PMC10143966

[68] Jannière L et al. 2007 Genetic evidence for a link between glycolysis and DNA replication. PLoS ONE **2**, e447. (10.1371/journal.pone.0000447) 17505547 PMC1866360

[69] Nouri H et al. 2018 Multiple links connect central carbon metabolism to DNA replication initiation and elongation in *Bacillus subtilis*. DNA Res. **25**, 641-653. (10.1093/dnares/dsy031) 30256918 PMC6289782

[70] Maciąg M, Nowicki D, Janniere L, Szalewska-Pałasz A, Węgrzyn G. 2011 Genetic response to metabolic fluctuations: correlation between central carbon metabolism and DNA replication in *Escherichia coli*. Microb. Cell Factories. **10**, 19. (10.1186/1475-2859-10-19) PMC3080795 21453533

[71] Maciąg-Dorszyńska M, Ignatowska M, Jannière L, Węgrzyn G, Szalewska-Pałasz A. 2012 Mutations in central carbon metabolism genes suppress defects in nucleoid position and cell division of replication mutants in *Escherichia coli*. Gene **503**, 31-35. (10.1016/j.gene.2012.04.066) 22565187

[72] Krause K et al. 2020 The role of metabolites in the link between DNA replication and central carbon metabolism in *Escherichia coli*. Genes **11**, 447. (10.3390/genes11040447) 32325866 PMC7231150

[73] Bergé M et al. 2020 Bacterial cell cycle control by citrate synthase independent of enzymatic activity. eLife **9**, e52272. (10.7554/eLife.52272) 32149608 PMC7083601

[74] Boukouris AE, Zervopoulos SD, Michelakis ED. 2016 Metabolic enzymes moonlighting in the nucleus: metabolic regulation of gene transcription. Trends Biochem. Sci. **41**, 712-730. (10.1016/j.tibs.2016.05.013) 27345518

[75] Li X, Egervari G, Wang Y, Berger SL, Lu Z. 2018 Regulation of chromatin and gene expression by metabolic enzymes and metabolites. Nat. Rev. Mol. Cell Biol. **19**, 563-578. (10.1038/s41580-018-0029-7) 29930302 PMC6907087

[76] Snaebjornsson MT, Schulze A. 2018 Non-canonical functions of enzymes facilitate cross-talk between cell metabolic and regulatory pathways. Exp. Mol. Med. **50**, 1-16. (10.1038/s12276-018-0065-6) PMC5938058 29657328

[77] Vogelauer M, Rubbi L, Lucas I, Brewer BJ, Grunstein M. 2002 Histone acetylation regulates the time of replication origin firing. Mol. Cell **10**, 1223-1233. (10.1016/S1097-2765(02)00702-5) 12453428

[78] Cai L, Sutter BM, Li B, Tu BP. 2011 Acetyl-CoA induces cell growth and proliferation by promoting the acetylation of histones at growth genes. Mol. Cell **42**, 426-437. (10.1016/j.molcel.2011.05.004) 21596309 PMC3109073

[79] Sutendra G et al. 2014 A nuclear pyruvate dehydrogenase complex is important for the generation of acetyl-CoA and histone acetylation. Cell **158**, 84-97. (10.1016/j.cell.2014.04.046) 24995980

[80] Wellen KE, Hatzivassiliou G, Sachdeva UM, Bui TV, Cross JR, Thompson CB. 2009 ATP-citrate lyase links cellular metabolism to histone acetylation. Science **324**, 1076-1080. (10.1126/science.1164097) 19461003 PMC2746744

[81] Zheng L, Roeder RG, Luo Y. 2003 S phase activation of the histone H2B promoter by OCA-S, a coactivator complex that contains GAPDH as a key component. Cell **114**, 255-266. (10.1016/S0092-8674(03)00552-X) 12887926

[82] Dai RP et al. 2008 Histone 2B (H2B) expression is confined to a proper NAD+/NADH redox status. J. Biol. Chem. **283**, 26 894-26 901. (10.1074/jbc.M804307200) 18682386

[83] Ma R et al. 2019 Exogenous pyruvate represses histone gene expression and inhibits cancer cell proliferation via the NAMPT–NAD+–SIRT1 pathway. Nucleic Acids Res. **47**, 11 132-11 150. (10.1093/nar/gkz864) PMC6868375 31598701

[84] Devbhandari S, Jiang J, Kumar C, Whitehouse I, Remus D. 2017 Chromatin constrains the initiation and elongation of DNA replication. Mol. Cell **65**, 131-141. (10.1016/j.molcel.2016.10.035) 27989437 PMC5256687

[85] Kurat CF, Yeeles JTP, Patel H, Early A, Diffley JFX. 2017 Chromatin controls DNA replication origin selection, lagging-strand synthesis, and replication fork rates. Mol. Cell **65**, 117-130. (10.1016/j.molcel.2016.11.016) 27989438 PMC5222724

[86] Bellush JM, Whitehouse I. 2017 DNA replication through a chromatin environment. Phil. Trans. R. Soc. B **372**, 20160287. (10.1098/rstb.2016.0287) 28847824 PMC5577465

[87] Sivanand S, Viney I, Wellen KE. 2018 Spatiotemporal control of acetyl-CoA metabolism in chromatin regulation. Trends Biochem. Sci. **43**, 61-74. (10.1016/j.tibs.2017.11.004) 29174173 PMC5741483

[88] Li X et al. 2018 Nuclear PGK1 alleviates ADP-dependent inhibition of CDC7 to promote DNA replication. Mol. Cell **72**, 650-660. (10.1016/j.molcel.2018.09.007) 30392930

[89] Grosse F, Nasheuer HP, Scholtissek S, Schomburg U. 1986 Lactate dehydrogenase and glyceraldehyde-phosphate dehydrogenase are single-stranded DNA-binding proteins that affect the DNA-polymerase-alpha-primase complex. Eur. J. Biochem. **160**, 459-467. (10.1111/j.1432-1033.1986.tb10062.x) 3536507

[90] Popanda O, Fox G, Thielmann HW. 1998 Modulation of DNA polymerases *α*, *δ* and *ε* by lactate dehydrogenase and 3-phosphoglycerate kinase. Biochim. Biophys. Acta BBA - Gene Struct. Expr. **1397**, 102-117. (10.1016/S0167-4781(97)00229-7) 9545551

[91] Jindal HK, Vishwanatha JK. 1990 Functional identity of a primer recognition protein as phosphoglycerate kinase. J. Biol. Chem. **265**, 6540-6543. (10.1016/S0021-9258(19)39179-3) 2324090

[92] Konieczna A, Szczepańska A, Sawiuk K, Węgrzyn G, Łyżeń R. 2015 Effects of partial silencing of genes coding for enzymes involved in glycolysis and tricarboxylic acid cycle on the enterance of human fibroblasts to the S phase. BMC Cell Biol. **16**, 16. (10.1186/s12860-015-0062-8) 26017754 PMC4446904

[93] Fornalewicz K, Wieczorek A, Węgrzyn G, Łyżeń R. 2017 Silencing of the pentose phosphate pathway genes influences DNA replication in human fibroblasts. Gene **635**, 33-38. (10.1016/j.gene.2017.09.005) 28887160

[94] Wieczorek A, Fornalewicz K, Mocarski Ł, Łyżeń R, Węgrzyn G. 2018 Double silencing of relevant genes suggests the existence of the direct link between DNA replication/repair and central carbon metabolism in human fibroblasts. Gene **650**, 1-6. (10.1016/j.gene.2018.01.068) 29407228

[95] Schormann N, Hayden KL, Lee P, Banerjee S, Chattopadhyay D. 2019 An overview of structure, function, and regulation of pyruvate kinases. Protein Sci. **28**, 1771-1784. (10.1002/pro.3691) 31342570 PMC6739817

[96] Morgan HP, Zhong W, McNae IW, Michels PAM, Fothergill-Gilmore LA, Walkinshaw MD. 2014 Structures of pyruvate kinases display evolutionarily divergent allosteric strategies. R. Soc. Open Sci. **1**, 140120. (10.1098/rsos.140120) 26064527 PMC4448766

[97] Nguyen CC, Saier MH. 1995 Phylogenetic analysis of the putative phosphorylation domain in the pyruvate kinase of *Bacillus stearothermophilus*. Res Microbiol. **146**, 713-719. (10.1016/0923-2508(96)81067-9) 8584793

[98] Herzberg O et al. 1996 Swiveling-domain mechanism for enzymatic phosphotransfer between remote reaction sites. Proc. Natl Acad. Sci. USA **93**, 2652-2657. (10.1073/pnas.93.7.2652) 8610096 PMC39685

[99] Minges A, Ciupka D, Winkler C, Höppner A, Gohlke H, Groth G. 2017 Structural intermediates and directionality of the swiveling motion of pyruvate phosphate dikinase. Sci Rep. **7**, 45389. (10.1038/srep45389) 28358005 PMC5371819

[100] Alpert CA, Frank R, Stueber K, Deutscher J, Hengstenberg W. 1985 Phosphoenolpyruvate-dependent protein kinase enzyme I of *Streptococcus faecalis*: purification and properties of the enzyme and characterization of its active center. Biochemistry **24**, 959-964. (10.1021/bi00325a023) 3922407

[101] Teplyakov A et al. 2006 Structure of phosphorylated enzyme I, the phosphoenolpyruvate:sugar phosphotransferase system sugar translocation signal protein. Proc. Natl Acad. Sci. USA **103**, 16 218-16 223. (10.1073/pnas.0607587103) PMC1618308 17053069

[102] Goss NH, Evans CT, Wood HG. 1980 Pyruvate phosphate dikinase: sequence of the histidyl peptide, the pyrophosphoryl and phosphoryl carrier. Biochemistry **19**, 5805-5809. (10.1021/bi00566a022) 6257292

[103] Tolentino R, Chastain C, Burnell J. 2013 Identification of the amino acid involved in the regulation of bacterial pyruvate, orthophosphate dikinase and phosphoenolpyruvate synthetase. Adv. Biol. Chem. **03**, 12-21. (10.4236/abc.2013.33A003)

[104] Burnell JN, Chastain CJ. 2006 Cloning and expression of maize-leaf pyruvate, Pi dikinase regulatory protein gene. Biochem. Biophys. Res. Commun. **345**, 675-680. (10.1016/j.bbrc.2006.04.150) 16696949

[105] Burnell JN. 2010 Cloning and characterization of *Escherichia coli* DUF299: a bifunctional ADP-dependent kinase: Pi-dependent pyrophosphorylase from bacteria. BMC Biochem. **11**, 1. (10.1186/1471-2091-11-1) 20044937 PMC2817694

[106] Burnell JN, Hatch MD. 1984 Regulation of C4 photosynthesis: identification of a catalytically important histidine residue and its role in the regulation of pyruvate, Pi dikinase. Arch. Biochem. Biophys. **231**, 175-182. (10.1016/0003-9861(84)90375-8) 6326674

[107] Sakai H. 2004 Possible structure and function of the extra C-terminal sequence of pyruvate kinase from *Bacillus stearothermophilus*. J. Biochem. **136**, 471-476. (10.1093/jb/mvh152) 15625316

[108] Suzuki K, Ito S, Shimizu-Ibuka A, Sakai H. 2008 Crystal structure of pyruvate kinase from *Geobacillus stearothermophilus*. J. Biochem. **144**, 305-312. (10.1093/jb/mvn069) 18511452

[109] Jumper J et al. 2021 Highly accurate protein structure prediction with AlphaFold. Nature **596**, 583-589. (10.1038/s41586-021-03819-2) 34265844 PMC8371605

[110] Varadi M et al. 2022 AlphaFold protein structure database: massively expanding the structural coverage of protein-sequence space with high-accuracy models. Nucleic Acids Res. **50**, D439-D444. (10.1093/nar/gkab1061) 34791371 PMC8728224

[111] Sakai H, Suzuki K, Imahori K. 1986 Purification and properties of pyruvate kinase from *Bacillus stearothermophilus*. J. Biochem. **99**, 1157-1167. (10.1093/oxfordjournals.jbchem.a135579) 3711058

[112] Sanders GM, Dallmann HG, McHenry CS. 2010 Reconstitution of the B. subtilis replisome with 13 proteins including two distinct replicases. Mol. Cell **37**, 273-281. (10.1016/j.molcel.2009.12.025) 20122408

[113] Li Y, Chen Z, Matthews LA, Simmons LA, Biteen JS. 2019 Dynamic exchange of two essential DNA polymerases during replication and after fork arrest. Biophys J. **116**, 684-693. (10.1016/j.bpj.2019.01.008) 30686488 PMC6382952

[114] Hernández-Tamayo R, Oviedo-Bocanegra LM, Fritz G, Graumann PL. 2019 Symmetric activity of DNA polymerases at and recruitment of exonuclease ExoR and of PolA to the Bacillus subtilis replication forks. Nucleic Acids Res. **47**, 8521-8536. (10.1093/nar/gkz554) 31251806 PMC6895272

[115] Hanahan D, Weinberg RA. 2011 Hallmarks of cancer: the next generation. Cell **144**, 646-674. (10.1016/j.cell.2011.02.013) 21376230

[116] Macheret M, Halazonetis TD. 2015 DNA replication stress as a hallmark of cancer. Annu. Rev. Pathol. Mech. Dis. **10**, 425-448. (10.1146/annurev-pathol-012414-040424) 25621662

[117] Kotsantis P, Petermann E, Boulton SJ. 2018 Mechanisms of oncogene-induced replication stress: jigsaw falling into place. Cancer Discov. **8**, 537-555. (10.1158/2159-8290.CD-17-1461) 29653955 PMC5935233

[118] Petropoulos M, Champeris Tsaniras S, Taraviras S, Lygerou Z. 2019 Replication licensing aberrations, replication stress, and genomic instability. Trends Biochem. Sci. **44**, 752-764. (10.1016/j.tibs.2019.03.011) 31054805

[119] Primo LMF, Teixeira LK. 2020 DNA replication stress: oncogenes in the spotlight. Genet Mol. Biol. **43**(1 suppl. 1), e20190138. (10.1590/1678-4685-gmb-2019-0138) PMC7197996 31930281

[120] Vander Heiden MG, DeBerardinis RJ. 2017 Understanding the intersections between metabolism and cancer biology. Cell **168**, 657-669. (10.1016/j.cell.2016.12.039) 28187287 PMC5329766

[121] Martínez-Reyes I, Chandel NS. 2021 Cancer metabolism: looking forward. Nat. Rev. Cancer **21**, 669-680. (10.1038/s41568-021-00378-6) 34272515

[122] Hanahan D, Weinberg RA. 2000 The hallmarks of cancer. Cell **100**, 57-70. (10.1016/S0092-8674(00)81683-9) 10647931

[123] Loeb LA. 2011 Human cancers express mutator phenotypes: origin, consequences and targeting. Nat. Rev. Cancer. **11**, 450-457. (10.1038/nrc3063) 21593786 PMC4007007

[124] Hu CM et al. 2019 High glucose triggers nucleotide imbalance through O-GlcNAcylation of key enzymes and induces KRAS mutation in pancreatic cells. Cell Metab. **29**, 1334-1349. (10.1016/j.cmet.2019.02.005) 30853214

[125] Moretton A, Loizou JI. 2020 Interplay between cellular metabolism and the DNA damage response in cancer. Cancers **12**, 2051. (10.3390/cancers12082051) 32722390 PMC7463900

[126] Sobanski T, Rose M, Suraweera A, O'Byrne K, Richard DJ, Bolderson E. 2021 Cell metabolism and DNA repair pathways: implications for cancer therapy. Front. Cell Dev. Biol. **9**, 633305. (10.3389/fcell.2021.633305) 33834022 PMC8021863

[127] Chen Z, Odstrcil EA, Tu BP, McKnight SL. 2007 Restriction of DNA replication to the reductive phase of the metabolic cycle protects genome integrity. Science **316**, 1916-1919. (10.1126/science.1140958) 17600220

[128] Wikoff WR et al. 2009 Metabolomics analysis reveals large effects of gut microflora on mammalian blood metabolites. Proc. Natl Acad. Sci. USA **106**, 3698-3703. (10.1073/pnas.0812874106) 19234110 PMC2656143

[129] Pedersen HK et al. 2016 Human gut microbes impact host serum metabolome and insulin sensitivity. Nature **535**, 376-381. (10.1038/nature18646) 27409811

[130] Moriya T, Satomi Y, Murata S, Sawada H, Kobayashi H. 2017 Effect of gut microbiota on host whole metabolome. Metabolomics **13**, 101. (10.1007/s11306-017-1240-9)

[131] Bar N et al. 2020 A reference map of potential determinants for the human serum metabolome. Nature **588**, 135-140. (10.1038/s41586-020-2896-2) 33177712

[132] Wilmanski T et al. 2019 Blood metabolome predicts gut microbiome α-diversity in humans. Nat. Biotechnol. **37**, 1217-1228. (10.1038/s41587-019-0233-9) 31477923

[133] Fan Y, Pedersen O. 2021 Gut microbiota in human metabolic health and disease. Nat. Rev. Microbiol. **19**, 55-71. (10.1038/s41579-020-0433-9) 32887946

[134] Diener C et al. 2022 Genome–microbiome interplay provides insight into the determinants of the human blood metabolome. Nat. Metab. **4**, 1560-1572. (10.1038/s42255-022-00670-1) 36357685 PMC9691620

[135] Dekkers KF et al. 2022 An online atlas of human plasma metabolite signatures of gut microbiome composition. Nat. Commun. **13**, 5370. (10.1038/s41467-022-33050-0) 36151114 PMC9508139

[136] Krautkramer KA, Rey FE, Denu JM. 2017 Chemical signaling between gut microbiota and host chromatin: what is your gut really saying? J. Biol. Chem. **292**, 8582-8593. (10.1074/jbc.R116.761577) 28389558 PMC5448088

[137] Allen J, Sears CL. 2019 Impact of the gut microbiome on the genome and epigenome of colon epithelial cells: contributions to colorectal cancer development. Genome Med. **11**, 11. (10.1186/s13073-019-0621-2) 30803449 PMC6388476

[138] Cullin N, Azevedo Antunes C, Straussman R, Stein-Thoeringer CK, Elinav E. 2021 Microbiome and cancer. Cancer Cell **39**, 1317-1341. (10.1016/j.ccell.2021.08.006) 34506740

[139] Sirover MA. 1999 New insights into an old protein: the functional diversity of mammalian glyceraldehyde-3-phosphate dehydrogenase. Biochim. Biophys. ActaProtein Struct. Mol. Enzymol. **1432**, 159-184. 10.1016/s0167-4838(99)00119-3 10407139

[140] Sirover MA. 2011 On the functional diversity of glyceraldehyde-3-phosphate dehydrogenase: biochemical mechanisms and regulatory control. Biochim. Biophys. Acta BBA—Gen. Subj. **1810**, 741-751. (10.1016/j.bbagen.2011.05.010) 21640161

[141] Kim J, Dang CV. 2005 Multifaceted roles of glycolytic enzymes. Trends Biochem. Sci. **30**, 142-150. (10.1016/j.tibs.2005.01.005) 15752986

[142] Henderson B, Martin A. 2011 Bacterial virulence in the moonlight: multitasking bacterial moonlighting proteins are virulence determinants in infectious disease. Andrews-Polymenis HL, editor. Infect. Immun. **79**, 3476-3491. (10.1128/IAI.00179-11) 21646455 PMC3165470

[143] Konieczna A, Szczepańska A, Sawiuk K, Łyżeń R, Węgrzyn G. 2015 Enzymes of the central carbon metabolism: are they linkers between transcription, DNA replication, and carcinogenesis? Med. Hypotheses. **84**, 58-67. (10.1016/j.mehy.2014.11.016) 25491416

[144] Chuang C, Prasanth KR, Nagy PD. 2017 The glycolytic pyruvate kinase is recruited directly into the viral replicase complex to generate ATP for RNA synthesis. Cell Host Microbe. **22**, 639-652. (10.1016/j.chom.2017.10.004) 29107644

[145] Yu X, Li S. 2017 Non-metabolic functions of glycolytic enzymes in tumorigenesis. Oncogene **36**, 2629-2636. (10.1038/onc.2016.410) 27797379

[146] Lu Z, Hunter T. 2018 Metabolic kinases moonlighting as protein kinases. Trends Biochem. Sci. **43**, 301-310. (10.1016/j.tibs.2018.01.006) 29463470 PMC5879014

[147] Zecchini V et al. 2023 Fumarate induces vesicular release of mtDNA to drive innate immunity. Nature **615**, 499-506. 36890229 10.1038/s41586-023-05770-w PMC10017517

